# Altered effective connectivity in the emotional network induced by immersive virtual reality rehabilitation for post-stroke depression

**DOI:** 10.3389/fnhum.2022.974393

**Published:** 2022-08-02

**Authors:** Jia-Jia Wu, Mou-Xiong Zheng, Xu-Yun Hua, Dong Wei, Xin Xue, Yu-Lin Li, Xiang-Xin Xing, Jie Ma, Chun-Lei Shan, Jian-Guang Xu

**Affiliations:** ^1^Department of Rehabilitation Medicine, Yueyang Hospital of Integrated Traditional Chinese and Western Medicine, Shanghai University of Traditional Chinese Medicine, Shanghai, China; ^2^Engineering Research Center of Traditional Chinese Medicine Intelligent Rehabilitation, Ministry of Education, Shanghai, China; ^3^Department of Traumatology and Orthopedics, Yueyang Hospital of Integrated Traditional Chinese and Western Medicine, Shanghai University of Traditional Chinese Medicine, Shanghai, China; ^4^School of Rehabilitation Science, Shanghai University of Traditional Chinese Medicine, Shanghai, China

**Keywords:** post-stroke depression (PSD), effective connectivity, granger causality analysis (GCA), immersive virtual reality, emotional network

## Abstract

Post-stroke depression (PSD) is a serious complication of stroke that significantly restricts rehabilitation. The use of immersive virtual reality for stroke survivors is promising. Herein, we investigated the effects of a novel immersive virtual reality training system on PSD and explored induced effective connectivity alterations in emotional networks using multivariate Granger causality analysis (GCA). Forty-four patients with PSD were equally allocated into an immersive-virtual reality group and a control group. In addition to their usual rehabilitation treatments, the participants in the immersive-virtual reality group participated in an immersive-virtual reality rehabilitation program, while the patients in the control group received 2D virtual reality rehabilitation training. The Hamilton Depression Rating Scale, modified Barthel Index (MBI), and resting-state functional magnetic resonance imaging (rsfMRI) data were collected before and after a 4-week intervention. rsfMRI data were analyzed using multivariate GCA. We found that the immersive virtual reality training was more effective in improving depression in patients with PSD but had no statistically significant improvement in MBI scores compared to the control group. The GCA showed that the following causal connectivities were strengthened after immersive virtual reality training: from the amygdala, insula, middle temporal gyrus, and caudate nucleus to the dorsolateral prefrontal cortex; from the insula to the medial prefrontal cortex; and from the thalamus to the posterior superior temporal sulcus. These causal connectivities were weakened after treatment in the control group. Our results indicated the neurotherapeutic use of immersive virtual reality rehabilitation as an effective non-pharmacological intervention for PSD; the alteration of causal connectivity in emotional networks might constitute the neural mechanisms underlying immersive-virtual reality rehabilitation in PSD.

## Introduction

Patients after stroke are at an increased risk of impaired mental health (Medeiros et al., 2020). Depression affects around 30−60% of stroke survivors, manifesting as pessimism, despair, sleep disorders, and inferiority (Hackett and Pickles, 2014). Despite the fact that post-stroke depression (PSD) is widespread and linked to other deficits, concurrent psychological issues are often ignored or undiagnosed. Studies have shown that patients with PSD had less interest, reduced motivation, and lower adherence to rehabilitation activities, resulting in poor functional outcomes (Loubinoux et al., 2012; Medeiros et al., 2020). Thus, in clinical practice, it is essential to emphasize the screening and intervention of PSD.

Recently, virtual reality (VR) has emerged as a valuable approach in the rehabilitation of stroke survivors (Laver et al., 2017). In fact, most conventional rehabilitation methodologies focus on mechanical and repetitive exercises, for which patients often lack interest and motivation. Thus, combining video games and physical therapy with a VR rehabilitation system may increase patient motivation and improve their physical function (Perrochon et al., 2019). According to previous studies, VR rehabilitation has been shown to enhance walking speed, balance, and upper limb function in patients after stroke (Zhang B. et al., 2021; Mugisha et al., 2022). These improvements might be associated with VR’s ability to create conditions that promote meaningful repeated practice, provide immediate feedback, and allow rehabilitation activities to be integrated into a more ecologically effective environment (Levin et al., 2015). In addition, VR has the potential to shift participants’ perspectives and break through the rigidity of the psychological experience (Perez-Marcos, 2018; Keshner et al., 2019). This ability has also been applied to treating various emotional disorders (Fernández-Caballero et al., 2017). The effect of VR-based therapy on depression symptoms in patients with chronic stroke has been demonstrated in recent years (Zhang Q. et al., 2021). However, the patients involved in those prior investigations almost all experienced depression symptoms but were not diagnosed with PSD. Few relevant studies on immersive-VR have been found in patients diagnosed with PSD.

Post-stroke depression is also associated with anomalies in brain structure and function, somewhat similar to major depressive disorder (MDD) (Pan et al., 2022). Many neuroimaging studies have advanced our knowledge of the neural mechanisms of PSD. To explain the networks involved in PSD, most researchers have focused on a ventromedial prefrontal cortex−anterior cingulate cortex−amygdala−thalamus emotional circuit, which includes changes in the structure, functional connection, excitability, and causality of the thalamus, anterior cingulate cortex, prefrontal cortex (PFC), amygdala (Amyg), insula (INS), temporal pole, hippocampus, and basal ganglia (BG) (Shi et al., 2019; Zhang et al., 2019). However, there is little research on the functional brain alterations induced by immersive VR rehabilitation in the treatment of PSD.

In the present study, we presented a novel immersive VR training system, tested it on PSD patients, and compared the effects on depression to conventional 2D rehabilitation activities. It was hypothesized that rehabilitation combined with immersive VR training would improve depressive outcomes. Furthermore, we investigated alterations in effective connectivity in emotional networks after immersive VR rehabilitation for PSD using multivariate Granger causality analysis (GCA). GCA is a powerful technique for extracting such connectivity from data, which provide sensitive evidence for the detection of functional interactions between brain regions.

## Materials and methods

### Participants

We included participants who: met the Diagnostic and Statistical Manual of Mental Disorders criteria for depressive disorder with a Hamilton depression Rating Scale (HAMD) score of 17 or higher; met the criteria for the diagnosis of cerebral infarction; were within 2 weeks to 12 months after stroke; signed informed consent; were 18–65 years old; were right-handed. There were no limits on patient sex. Experienced neuropsychologists conducted clinical interviews. The exclusion criteria included: (a) history of other severe mental disorders, epilepsy, drug abuse, or antidepressant use at stroke onset; (b) serious systemic diseases such as diabetes and uremia; (c) vision disorders and severe motor dysfunction that could interfere with the execution of the VR training task; (d) serious negative suicidal ideation; and (f) inability to cooperate and complete training and evaluation for any reason.

**FIGURE 1:**
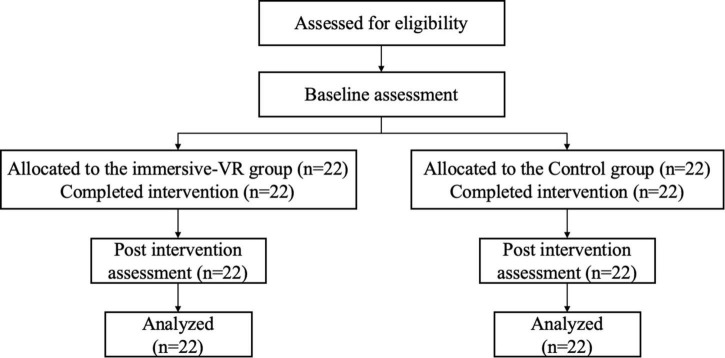
The flow diagram of the study process. VR, virtual reality.

The research was performed in rehabilitation clinics in Yueyang Hospital, affiliated with Shanghai University of Traditional Chinese Medicine. The study protocol was approved by the local research ethics committee and registered with the Chinese Clinical Trial Registry (ChiCTR2100052132). There were 44 patients with PSD included in the study. They were allocated to two groups: the immersive-VR group (*n* = 22) and the control group (*n* = 22). Demographics (i.e., age, gender, and duration of stroke), HAMD, modified Barthel Index (MBI), and resting-state functional MRI (rsfMRI) data were collected from each participant. The study process is detailed in Figure 1.

### Intervention

In addition to their usual rehabilitation treatments such as physical and occupational therapy, which mostly involved motor training, the participants in the immersive-VR group underwent an immersive-VR rehabilitation training program for 20 min/d, 5 days a week for 4 weeks, while the patients in the control group underwent 2D VR rehabilitation training.

#### Immersive virtual reality rehabilitation

In the immersive-VR group, 3D immersive-VR rehabilitation was conducted by the rehabilitation system developed by Shanghai University of Traditional Chinese Medicine in collaboration with the School of Mechatronic Engineering and Automation of Shanghai University. The virtual environment was developed and rendered using the Unity 3D engine and was run on a PC (CPU Intel I7 9700K). A VR headset (VIVE Pro; HTC, Taoyuan City, Taiwan) was used to present the 3D virtual training environment in the rehabilitation system. Participants could rotate their heads for a 360° view of the virtual scene and interact freely in the virtual environment with the VR headset. Assisted by a therapist, participants were asked to sit in an open room and wear a VR helmet. Through the headset, the participants could view a bright and spacious living room that could make them comfortable and stable. Within the virtual environment, the indoor furniture layout restored reality and provided the participants with an immersive training experience. A built-in positioning function in the software was conducted firstly to correspond the sofa position in the scene to the patient’s sitting position. During the training task, three different common fruits were generated in the virtual scene, and participants were asked to select the fruit prompted by sound or text within the virtual scene. After completing the interaction, encouraging vocal feedback was provided to the participants.

**FIGURE 2:**
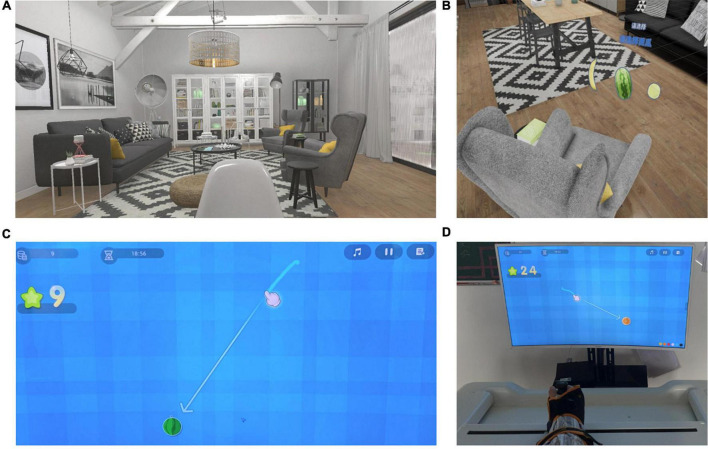
The virtual reality (VR) training scenarios in the immersive-VR group and the control group. **(A,B)** The 3D virtual scenario shown through the VR helmet for immersive VR training in the immersive-VR group. **(C,D)** The 2D virtual scenario shown on the screen for VR training in the control group.

An evaluation score was given after all training tasks were completed. In the VR rehabilitation system, 3D upper limb reaching movements were captured and mapped onto the movements of a virtual arm. Before training, the difficulty of the training task was set according to the patient’s upper limb function to ensure that the fruit appeared within the patient’s reach. Participants were asked to select the fruits using a wireless remote control secured with straps if the affected upper limb function was sufficient to complete the harvesting task. Assistance with a manipulator was be provided if the patients could not complete the harvesting task by themselves. The immersive VR scene is presented in Figure 2.

#### 2D virtual reality rehabilitation

In the control group, 2D VR rehabilitation was conducted using the Fourier M2 rehabilitation system (Fourier International Pte. Ltd., Shanghai, China). Assisted by a therapist, participants were asked to sit on a chair; the paretic hand was secured on the end-effector with straps. Through the end-effector, the upper limb movements could be captured. The user’s movements through the end-effector were mapped onto the movements of a virtual hand on the computer screen. A harvesting task was selected for the patients in the control group. Participants were asked to reach the fruits displayed on the computer screen through the virtual hand during the training task. Before training, the difficulty of the game was set according to the patient’s upper limb function. The 2D VR scene is presented in Figure 2.

### Outcome measures

All outcome measures were evaluated before and after a 4-week rehabilitation. A trained investigator administered the clinical measurements, and the rsfMRI data was analyzed by another trained investigator. In this study, both investigators were blinded to the group allocations.

#### Clinical assessments

In this study, HAMD score was considered the primary outcome measure. It is the most commonly used clinical evaluation scale for depression, which is frequently applied in patients with PSD (Lin et al., 2020). Higher scores indicate a stronger depressive symptoms.

The secondary outcomes concerned the effects on the activities of daily living and effective connectivity in the brain emotional networks.

The MBI is frequently used as a measure of activities of daily living. The scale comprises 10 items: feeding, bowel control, bladder control, personal hygiene, transfer, dressing, toileting, ambulation, bathing, and stair climbing. The total score ranges from 0 (completely dependent) to 100 (fully independent).

#### MRI scanning

MRI measurements were conducted on a 3.0-Tesla Magnetom Verio MRI scanner (Siemens Healthcare, Erlangen, Germany) with a 32-channel phased-array head coil. All participants were asked to stay awake and still without thinking during scanning, and foam pillows were used to minimize head movement. The rsfMRI data were acquired by a single-shot gradient-recalled echo-planar imaging sequence with the following parameters: repetition time = 3,000 ms, flip angle = 90°, interleaved scanning order, slice number = 43, matrix size = 64 × 64, slice thickness = 3.0 mm (no-gap), field of view = 230 mm × 230 mm, and 200 volumes. T1-weighted data were acquired by a 3D magnetization-prepared rapid acquisition gradient-echo sequences with the following parameters: repetition time = 1,900 ms, inversion time = 900 ms, echo time = 2.93 ms, flip angle = 9°, field of view = 256 mm × 256 mm, slice thickness = 1 mm, acquisition matrix = 256 × 256, and number of averages = 1.

#### Pre-processing of resting-state functional magnetic resonance imaging data

The rsfMRI data was pre-processed using Statistical Parametric Mapping software (version 12; Wellcome Trust Centre for Neuroimaging, London, United Kingdom)^1^ in the MATLAB platform (*v.* 2014a; MathWorks, Natick, MA, United States). Data pre-processing steps included the following: removal of the first 10 volumes; slice timing correction; realignment for head motion correction; spatial normalization to the Montreal Neurological Institute template (resampled voxels to 3 mm × 3 mm × 3 mm); smoothing with a 6-mm full-width at half-maximum Gaussian kernel; detrending; and filtering (0.01–0.08 Hz).

#### Effective connectivity analysis with resting-state functional magnetic resonance imaging

**FIGURE 3:**
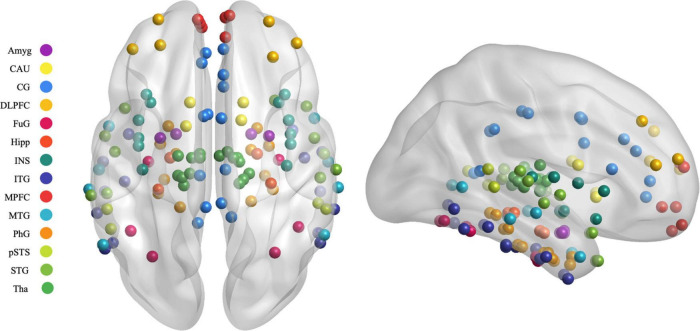
Map illustrating brain of the regions of interest selected in the granger causality analysis. MPFC, medial prefrontal cortex; DLPFC, dorsolateral prefrontal cortex; SFG, superior frontal gyrus; MFG, middle frontal gyrus; OrG, orbital gyrus; STG, superior temporal gyrus; MTG, middle temporal gyrus; ITG, inferior temporal gyrus; FuG, fusiform gyrus; PhG, parahippocampal gyrus; pSTS, posterior superior temporal sulcus; INS, insular gyrus; CG, cingulate gyrus; Amyg, amygdala; Hipp, hippocampus; BG, basal ganglia; CAU, caudate, Tha, Thalamus.

Previous studies have shown some reliable findings that the prefrontal lobe−limbic system−cortical striatum−thalamic circuit was important in the emotional networks involved in PSD (Shi et al., 2019; Zhang et al., 2019). In the present study, 122 regions of interest (ROIs) in the emotional network were selected based on previous rsfMRI studies from 246 subregions using the latest human Brainnetome Atlas (Fan et al., 2016). The brain regions are shown in Figure 3 and Supplementary Table 1.

In this study, we used Granger causality to analyze the effective connectivity between the reference signals of the seed regions (Granger, 1969). Multivariate GCA was performed using the REST toolbox^2^ following a published method (Shi et al., 2019). For each participant, time series data of the selected ROI were extracted from the pre-processed data. These time series data for each participant were then entered into coefficient-based ROI-wise multivariate GCA computation. The resulting GCA signed-path coefficients characterized the strength and direction of the temporal relationships among the brain regions (Shi et al., 2019).

### Statistical analysis

SPSS (Version 21.0; IBM Corp., Armonk, NY, United States) and MATLAB 2014a were used to perform the statistical analyses in this study. The baseline characteristics of the two groups were compared using the Student’s *t*-test for continuous variables or Pearson’s χ^2^ test for categorical variables. To compare the effects in the two groups regarding HAMD scores, MBI scores, and GCA signed-path coefficients, Analysis of Variance (ANOVA) tests with repeated measures with a between-subject factor at two levels (two groups) and a within-subject factor at two levels (time: before and after treatment) were conducted. In addition, the Time × Group interaction effects were analyzed. When a significant interaction effect was detected, *post-hoc* analysis with Bonferroni correction was used. The statistical significance threshold was set at *p* < 0.05.

## Results

### Demographic and clinical characteristics

A total of 44 patients with PSD were included in the analysis: 22 patients in the immersive-VR group and 22 patients in the control group. No significant differences were found in terms of age, gender, days after stroke, side of the lesion, and HAMD scores between the two groups at baseline. The demographic and clinical characteristics are summarized in Table 1.

**TABLE 1:** Demographic and clinical characteristics.

Variable	The immersive-VR group	The control group	*p*-value
Age (years)	51.95 ± 12.41	50.73 ± 9.99	0.73
Gender (male/female)	13/9	14/8	0.76
Duration after stroke (months)	7.27 ± 3.79	6.82 ± 3.26	0.63
Side of lesion (left/right)	16/6	15/7	0.74
HAMD scores	23.09 ± 1.87	22.05 ± 1.94	0.12

VR, virtual reality; HAMD, Hamilton depression Rating Scale.

### Effect of immersive-virtual reality on depression

The Time × Group effect was significant for HAMD scores [*F*(1, 42) = 30.76, *p* < 0.001]. After treatment, only the immersive-VR group showed a significant reduction in HAMD scores compared to the baseline levels (*p* < 0.001). At baseline, there was no significant difference between the two groups. After the intervention, HAMD scores in the immersive-VR group were significantly lower than those in the control group (*p* < 0.001; Table 2).

**TABLE 2:** *Pre* and *post* outcome measures of the immersive-VR group and the control group.

Outcome	*F*-value (interaction)	*p*-value	The immersive-VR group			The control group		
			*Pre*	*Post*	*p*-value	*Pre*	*Post*	*p*-value
HAMD	30.76	<0.001	23.09 ± 1.87	18.27 ± 3.33	0.00	22.05 ± 1.94	21.09 ± 1.85	0.06
MBI	2.02	0.16	73.64 ± 18.91	78.86 ± 16.54	<0.001	74.55 ± 15.35	81.59 ± 13.75	<0.001

VR, virtual reality; HAMD, Hamilton depression Rating Scale; MBI, modified Barthel Index.

### Effect of immersive-virtual reality on modified Barthel index

At baseline, there was no significant difference in MBI between the two groups. After treatment, the two groups both showed significantly higher MBI scores compared to the baseline levels (*p* < 0.001). However, no Time × Group interaction was found in MBI [*F*(1, 42) = 2.02, *p* = 0.16; Table 2].

### Effective connectivity results in resting-state functional magnetic resonance imaging

**FIGURE 4:**
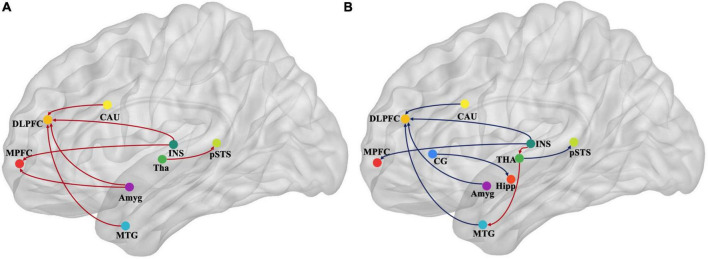
Map illustrating brain of multivariate GCA signed-path coefficients alteration after treatment in the immersive-VR group **(A)** and the control group **(B)**. Some brain regions only showed projection position. Blue/red arrows designate significantly weaken/strengthen of causal connectivity after treatment as compared to baseline levels. MPFC, medial prefrontal cortex; DLPFC, dorsolateral prefrontal cortex; MTG, middle temporal gyrus; pSTS, posterior superior temporal sulcus; INS, insular gyrus; CG, cingulate gyrus; Amyg, amygdala; Hipp, hippocampus; CAU, caudate, Tha, Thalamus. GCA, granger causality analysis.

**FIGURE 5:**
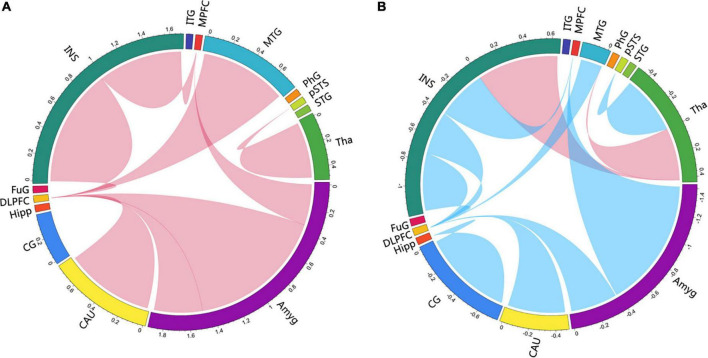
Circlize maps of signed-path coefficient alteration after treatment in the immersive-VR group **(A)** and the control group **(B)** from multivariate granger causality analysis. The parameters represent the intensity of the signed-path coefficients differences after treatment as compared to baseline levels. Blue/red lines designate significantly weaken/strengthen of causal connectivity after treatment as compared to baseline levels. MPFC, medial prefrontal cortex; DLPFC, dorsolateral prefrontal cortex; MTG, middle temporal gyrus; pSTS, posterior superior temporal sulcus; INS, insular gyrus; CG, cingulate gyrus; Amyg, amygdala; Hipp, hippocampus; CAU, caudate, Tha, Thalamus.

We used Granger causality to investigate alterations in brain effective connectivity in emotional networks. *Post-hoc* analysis with Bonferroni correction was used when a significant interaction effect was detected. The GCA results are presented in Table 3 and summarized in Figures 4, 5.

**TABLE 3:** *Pre* and *post* signed-path coefficients of multivariate granger causality analysis results showing significant Time × Group Interaction in the two groups.

ROI1	ROI2	*F*-value (interaction)	*p*-value	The immersive-VR group			The control group		
				*Pre*	*Post*	*p*-value	*Pre*	*Post*	*p*-value
Tha_R_8_4	pSTS_R_2_2	13.1626	0.0012	–0.1985	**0.2932**	0.0353	0.1310	**−0.3468**	0.0034
BG_R_6_1	MFG_R_7_3	12.6577	0.0014	–0.1451	0.6196	0.0139	0.2766	–0.2037	0.0204
MTG_L_4_4	MFG_L_7_3	12.6051	0.0014	–0.3213	0.4266	0.0381	0.1422	–0.0565	0.0042
INS_L_6_6	MFG_L_7_3	12.2226	0.0017	–0.2762	**0.7078**	0.0367	0.0658	–0.3920	0.0058
Amyg_L_2_2	MFG_R_7_3	12.1175	0.0017	–0.1749	**0.9664**	0.0056	0.1580	–0.2898	0.0896
Amyg_L_2_1	MFG_L_7_3	11.5652	0.0021	**0.4474**	**0.8533**	0.0194	**0.4888**	0.1183	0.0237
INS_L_6_3	Tha_L_8_5	11.1440	0.0025	–0.4270	–0.1809	0.0605	–0.3025	0.3370	0.0048
CG_L_7_4	Hipp_L_2_2	11.0412	0.0026	0.0587	**0.4399**	0.0936	0.3264	−**0.4113**	0.0023
INS_R_6_3	SFG_R_7_7	10.9122	0.0027	–0.1939	**0.5035**	0.0173	0.2837	–0.1126	0.0408
Tha_L_8_4	MTG_L_4_3	10.2591	0.0035	0.2524	–0.2244	0.1904	0.0348	0.4577	0.0009
INS_R_6_5	MFG_L_7_3	10.1095	0.0037	–0.3646	0.7435	0.0881	0.0584	–0.2839	0.0048
Amyg_L_2_2	MTG_R_4_2	9.8908	0.0040	0.3379	**0.7306**	0.0658	**0.7161**	–0.3253	0.0096
Amyg_L_2_1	SFG_R_7_7	9.8481	0.0041	**0.1964**	**0.5424**	0.0058	0.4969	0.1166	0.2551

Group mean path coefficients with predictions going from ROI1 to ROI2 are shown. Group means in bold are significantly different from zero (two-tailed tests; *p* ≤ 0.05). SFG, superior frontal gyrus; MFG, middle frontal gyrus; MTG, middle temporal gyrus; ITG, inferior temporal gyrus; pSTS, posterior superior temporal sulcus; INS, insular gyrus; CG, cingulate gyrus; Amyg, amygdala; Hipp, hippocampus; BG, basal ganglia; Tha, Thalamus. Bold values designate that group means are significantly different from zero.

After treatment in the immersive-VR group, the causal connectivity between the following regions was strengthened significantly compared to pre-treatment: from Tha_R_8_4 to pSTS_R_2_2, from BG_R_6_1 to MFG_R_7_3, from MTG_L_4_4 to MFG_R_7_3, from INS_L_6_6 to MFG_R_7_3, from Amyg_L_2_2 to MFG_R_7_3, from Amyg_L_2_1 to MFG_L_7_3, from INS_R_6_3 to SFG_R_7_7, and from Amyg_L_2_1 to SFG_R_7_7.

After treatment in the control group, the causal connectivity in patients with PSD from INS_L_6_3 to Tha_L_8_5 and from Tha_L_8_4 to MTG_L_4_3 was strengthened significantly. The causal connectivity between the following regions was weakened significantly compared to pre-treatment: from Tha_R_8_4 to pSTS_R_2_2, from BG_R_6_1 to MFG_R_7_3, from MTG_L_4_4 to MFG_R_7_3, from INS_L_6_6 to MFG_R_7_3, from Amyg_L_2_1 to MFG_L_7_3, from INS_R_6_3 to SFG_R_7_7, from CG_L_7_4 to Hipp_L_2_2, from INS_R_6_5 to MFG_L_7_3, and from Amyg_L_2_2 to MTG_R_4_2.

## Discussion

In this study, we found that immersive VR training was more effective in improving depression in patients with PSD but had no statistically significant improvements activities of daily living when compared to the control group. Furthermore, we used multivariate GCA to explore the alterations in effective connectivity in the brain areas of emotional networks. Notably, the GCA results mainly showed that the causal connectivity from the amygdala, INS, middle temporal gyrus (MTG), and caudate nucleus to the dorsolateral PFC (DLPFC); from the INS to medial PFC (MPFC); and from the thalamus to the posterior superior temporal sulcus were strengthened after immersive VR training, while they were weakened after treatment in the control group.

In the past few years, many preliminary studies have shown that VR-based training combined with traditional rehabilitation was more effective for improving depressive symptoms in stroke survivors than traditional rehabilitation (Gao et al., 2021). However, in most previous studies, the mood symptoms were not the primary therapeutic target, and the studied included patients with chronic stroke. Moreover, the enrolled stroke survivors of the previous studies had no definite diagnoses of depression or only had mild depressive symptoms. In the present study, we included stroke survivors with a wide range of disease durations (2 weeks to 12 months) and HAMD scores of more than 17. Consistent with previous studies, our results showed that immersive VR intervention led to a significant reduction in depressive symptoms in patients with PSD.

Brain remodeling is the core mechanism of stroke rehabilitation (Richards and Cramer, 2021). Regarding PSD, previous studies have already detected some reliable findings that the prefrontal lobe−limbic system−cortical striatum−thalamic circuit was important to explain the emotional network involvement in PSD (Shi et al., 2019; Zhang et al., 2019). Using multi-modal neuroimaging approaches, structural and functional remodeling were found in specific brain regions of the emotional networks, such as the thalamus, anterior cingulate cortex, PFC, temporal pole, and amygdala (Pandya et al., 2012). Moreover, the functional interrelations among structures of the emotional networks were also crucial to emotional regulation in PSD. It was demonstrated that patients with PSD demonstrated disrupted functional connectivity between specific brain regions of the emotional networks (Shi et al., 2019).

The basic neuroscience behind VR-based treatment has mainly been linked to functional brain remodeling (Feitosa et al., 2022). The present study also set the imaging ROIs in the emotional networks, as reported previously, to explore the functional remodeling induced by VR supporting emotion recovery. The interesting result from GCA in the present study was that some effective connectivities of the emotional networks increased significantly with the significant improvement of depression after immersive-VR rehabilitation. In contrast, those connectivities decreased significantly after treatment in the control group without significant improvement in depression. Among these, the DLPFC and MPFC were mainly directionally regulated. The PFC has long been thought to make critical contributions to emotional processing (Renner et al., 2015; Xu et al., 2020). The DLPFC has a wide range of neural connections and complex structural patterns and plays a vital role in emotional information processing, especially in negative emotional judgment (DeYoung et al., 2010). Activation of the DLPFC can increase negative emotional judgment and increase negative emotion transmission to other brain regions. Given the critical role of the DLPFC in emotional regulation, it is the most common neural modulation target of emotional disorders (Struckmann et al., 2022). The MPFC plays a critical role in reward processing, decision-making, and inhibiting negative emotional responses (Hänsel and von Känel, 2008; Rolls et al., 2020). It is also involved in the pathophysiology of MDD (Renner et al., 2015). The present study found an increased directional activation of the DLPFC and MPFC by brain regions of emotional networks after immersive VR rehabilitation; we speculated that VR might reduce the judgment and transmission of negative emotions by increasing the directional regulation of the DLPFC and MPFC, improving depression. Moreover, the decreased directional regulation of the DLPFC and MPFC in the control group might be a concomitant neural manifestation of no significant improvement in PSD symptoms.

The structures that showed altered effective connectivity with the DLPFC and MPFC were located in the caudate nucleus, insula, anterior superior temporal sulcus, and the amygdala. The affective control network, such as the thalamus, amygdala, insular, and striatum, has been shown to be involved with the emotional dysregulations in MDD (Li et al., 2022). Many studies observed that the functional activation and structural remodeling of the amygdala, which is a key component of the limbic system, might be the key indicators for the remission of depression (Lanteaume et al., 2007). As a primary hub of the frontal−limbic circuit, the insula is also associated with depression; people with depression may pay more attention to emotional subjective feelings induced by abnormal insular activation (Herbert and Pollatos, 2012; Vilares et al., 2012). Functional and structural abnormalities have also been found in thalamocortical networks in depression, which are crucial for cognitive and emotional processes (Timbie and Barbas, 2015). It was found that patients with temporal lobe, frontal lobe, and basal ganglia infarction were susceptible to PSD (Zhang et al., 2012). The temporal lobe is involved in emotion, memory, language, and other functions (Fang et al., 2017), and temporal lobe dysfunction is related to emotional disorders (Zhang et al., 2017). The present study found that the positive directional regulation of the DLPFC and MPFC by these brain regions of the emotional network played an important role in treating PSD. Moreover, strengthened connectivity from the amygdala to the MPFC in the immersive-VR group and weakened connectivity from the amygdala to the MTG and from the cingulate cortex to the hippocampus in the control group are consistent with this context. The findings also provided important guiding significance for the neural modulation of depression. It can be suggested that the modulation of the effective connectivity between specific brain regions might have a better rehabilitation effect than the typical single-target modulation of the DLPFC or MPFC.

We also found that causal connectivities from the insula to the thalamus and from the thalamus to the MTG were strengthened after treatment in the control group. Previous literature has shown that emotional networks mainly produce negative regulation signals *via* the thalamus to reduce the activity of the insula in depression (Craig et al., 2000; Shi et al., 2019). Thus, the insula−thalamus−MTG regulation was increased after treatment in the control group without significant improvement in depression, which is speculated to be related to strengthened negative regulation in emotional networks.

### Limitations

First, we only evaluated the patients’ depressive symptoms and did not measure recovery of motor function, although poor performance in motor activities can lead to depression in general (Kim et al., 2019). Considering that 4 weeks of rehabilitation treatment might have little difference in motor function between two groups with a wide range of disease durations, we did not further analyze the impact of motor rehabilitation on depression. Second, in accordance with previous studies, we functionally localized the structures associated with emotional networks that have been implicated reliably in depression. However, it has been consistently found that these structures are characterized by abnormal activation patterns in depression. Finally, there was no follow-up period in this study, and it was unclear whether the observed efficacy is long-lasting. Thus, future research should focus on increasing the sample size, prolonging the training duration, and adding a follow-up period to better evaluate the effects of immersive VR on PSD.

## Conclusion

Overall, this study revealed that immersive-VR rehabilitation could improve depression in patients with PSD by exposing patients to an enriched, immersive-VR environment. Furthermore, this finding was supported by increased effective connectivity in emotional networks mainly directed toward the DLPFC and MPFC, which also showed a significant decrease in the control group. Our results indicated the neurotherapeutic use of immersive-VR rehabilitation as an effective non-pharmacological intervention for PSD as well as alterations of causal connectivity in emotional networks that might contribution to symptom improvement.

## Data availability statement

The raw data supporting the conclusions of this article will be made available by the authors, without undue reservation.

## Ethics statement

The studies involving human participants were reviewed and approved by the Ethics Committee of Yueyang Hospital, affiliated with Shanghai University of Traditional Chinese Medicine. The patients/participants provided their written informed consent to participate in this study.

## Author contributions

J-GX, C-LS, and X-YH defined and designed this study, and interpreted the results. M-XZ and J-JW analyzed the data and wrote the manuscript. DW, Y-LL, and XX ran the intervention. JM and X-XX collected the data. All authors revised and approved the current version of the manuscript.

## Footnotes

1
http://www.fil.ion.ucl.ac.uk/spm/software/spm12/

2
http://restfmri.net/forum/rest
