# Supplementary material for: Altered effective connectivity in the emotional network induced by immersive virtual reality rehabilitation for post-stroke depression
Source: Front Hum Neurosci. 2022 Aug 2;16:974393. doi: 10.3389/fnhum.2022.974393 (PMC9378829; doi:10.3389/fnhum.2022.974393)
Supplement: Supplementary file 1 [file Table_1.DOCX]

Supplementary Material

# Supplementary Table 1. Regions of interest selected in the granger causality analysis.

| **ROI Num** | **Brain region** | **Label of**  **Brainnetome Atlas** | **Anatomical and modified Cyto-architectonic descriptions** |
| --- | --- | --- | --- |
| ROI-001 | MPFC | SFG_L_7_7 | A10m, medial area 10 |
| ROI-002 | MPFC | SFG_R_7_7 |  |
| ROI-003 | DLPFC | MFG_L_7_1 | A9/46d, dorsal area 9/46 |
| ROI-004 | DLPFC | MFG_R_7_1 |  |
| ROI-005 | DLPFC | MFG_L_7_3 | A46, area 46 |
| ROI-006 | DLPFC | MFG_R_7_3 |  |
| ROI-007 | DLPFC | MFG_L_7_4 | A9/46v, ventral area 9/46 |
| ROI-008 | DLPFC | MFG_R_7_4 |  |
| ROI-009 | MPFC | OrG_L_6_1 | A14m, medial area 14 |
| ROI-010 | MPFC | OrG_R_6_1 |  |
| ROI-011 | MPFC | OrG_L_6_4 | A11m, medial area 11 |
| ROI-012 | MPFC | OrG_R_6_4 |  |
| ROI-013 | STG | STG_L_6_1 | A38m, medial area 38 |
| ROI-014 | STG | STG_R_6_1 |  |
| ROI-015 | STG | STG_L_6_2 | A41/42, area 41/42 |
| ROI-016 | STG | STG_R_6_2 |  |
| ROI-017 | STG | STG_L_6_3 | TE1.0 and TE1.2 |
| ROI-018 | STG | STG_R_6_3 |  |
| ROI-019 | STG | STG_L_6_4 | A22c, caudal area 22 |
| ROI-020 | STG | STG_R_6_4 |  |
| ROI-021 | STG | STG_L_6_5 | A38l, lateral area 38 |
| ROI-022 | STG | STG_R_6_5 |  |
| ROI-023 | STG | STG_L_6_6 | A22r, rostral area 22 |
| ROI-024 | STG | STG_R_6_6 |  |
| ROI-025 | MTG | MTG_L_4_1 | A21c, caudal area 21 |
| ROI-026 | MTG | MTG_R_4_1 |  |
| ROI-027 | MTG | MTG_L_4_2 | A21r, rostral area 21 |
| ROI-028 | MTG | MTG_R_4_2 |  |
| ROI-029 | MTG | MTG_L_4_3 | A37dl, dorsolateral area37 |
| ROI-030 | MTG | MTG_R_4_3 |  |
| ROI-031 | MTG | MTG_L_4_4 | aSTS, anterior superior temporal sulcus |
| ROI-032 | MTG | MTG_R_4_4 |  |
| ROI-033 | ITG | ITG_L_7_1 | A20iv, intermediate ventral area 20 |
| ROI-034 | ITG | ITG_R_7_1 |  |
| ROI-035 | ITG | ITG_L_7_2 | A37elv, extreme lateroventral area37 |
| ROI-036 | ITG | ITG_R_7_2 |  |
| ROI-037 | ITG | ITG_L_7_3 | A20r, rostral area 20 |
| ROI-038 | ITG | ITG_R_7_3 |  |
| ROI-039 | ITG | ITG_L_7_4 | A20il, intermediate lateral area 20 |
| ROI-040 | ITG | ITG_R_7_4 |  |
| ROI-041 | ITG | ITG_L_7_5 | A37vl, ventrolateral area 37 |
| ROI-042 | ITG | ITG_R_7_5 |  |
| ROI-043 | ITG | ITG_L_7_6 | A20cl, caudolateral of area 20 |
| ROI-044 | ITG | ITG_R_7_6 |  |
| ROI-045 | ITG | ITG_L_7_7 | A20cv, caudoventral of area 20 |
| ROI-046 | ITG | ITG_R_7_7 |  |
| ROI-047 | FuG | FuG_L_3_1 | A20rv, rostroventral area 20 |
| ROI-048 | FuG | FuG_R_3_1 |  |
| ROI-049 | FuG | FuG_L_3_2 | A37mv, medioventral area37 |
| ROI-050 | FuG | FuG_R_3_2 |  |
| ROI-051 | FuG | FuG_L_3_3 | A37lv, lateroventral area37 |
| ROI-052 | FuG | FuG_R_3_3 |  |
| ROI-053 | PhG | PhG_L_6_1 | A35/36r, rostral area 35/36 |
| ROI-054 | PhG | PhG_R_6_1 |  |
| ROI-055 | PhG | PhG_L_6_2 | A35/36c, caudal area 35/36 |
| ROI-056 | PhG | PhG_R_6_2 |  |
| ROI-057 | PhG | PhG_L_6_3 | TL, area TL (lateral PPHC, posterior parahippocampal gyrus) |
| ROI-058 | PhG | PhG_R_6_3 |  |
| ROI-059 | PhG | PhG_L_6_4 | A28/34, area 28/34 (EC, entorhinal cortex) |
| ROI-060 | PhG | PhG_R_6_4 |  |
| ROI-061 | PhG | PhG_L_6_5 | TI, area TI (temporal agranular insular cortex) |
| ROI-062 | PhG | PhG_R_6_5 |  |
| ROI-063 | PhG | PhG_L_6_6 | TH, area TH (medial PPHC) |
| ROI-064 | PhG | PhG_R_6_6 |  |
| ROI-065 | pSTS | pSTS_L_2_1 | rpSTS, rostroposterior superior temporal sulcus |
| ROI-066 | pSTS | pSTS_R_2_1 |  |
| ROI-067 | pSTS | pSTS_L_2_2 | cpSTS, caudoposterior superior temporal sulcus |
| ROI-068 | pSTS | pSTS_R_2_2 |  |
| ROI-069 | INS | INS_L_6_1 | G, hypergranular insula |
| ROI-070 | INS | INS_R_6_1 |  |
| ROI-071 | INS | INS_L_6_2 | vIa, ventral agranular insula |
| ROI-072 | INS | INS_R_6_2 |  |
| ROI-073 | INS | INS_L_6_3 | dIa, dorsal agranular insula |
| ROI-074 | INS | INS_R_6_3 |  |
| ROI-075 | INS | INS_L_6_4 | vId/vIg, ventral dysgranular and granular insula |
| ROI-076 | INS | INS_R_6_4 |  |
| ROI-077 | INS | INS_L_6_5 | dIg, dorsal granular insula |
| ROI-078 | INS | INS_R_6_5 |  |
| ROI-079 | INS | INS_L_6_6 | dId, dorsal dysgranular insula |
| ROI-080 | INS | INS_R_6_6 |  |
| ROI-081 | CG | CG_L_7_1 | A23d, dorsal area 23 |
| ROI-082 | CG | CG_R_7_1 |  |
| ROI-083 | CG | CG_L_7_2 | A24rv, rostroventral area 24 |
| ROI-084 | CG | CG_R_7_2 |  |
| ROI-085 | CG | CG_L_7_3 | A32p, pregenual area 32 |
| ROI-086 | CG | CG_R_7_3 |  |
| ROI-087 | CG | CG_L_7_4 | A23v, ventral area 23 |
| ROI-088 | CG | CG_R_7_4 |  |
| ROI-089 | CG | CG_L_7_5 | A24cd, caudodorsal area 24 |
| ROI-090 | CG | CG_R_7_5 |  |
| ROI-091 | CG | CG_L_7_6 | A23c, caudal area 23 |
| ROI-092 | CG | CG_R_7_6 |  |
| ROI-093 | CG | CG_L_7_7 | A32sg, subgenual area 32 |
| ROI-094 | CG | CG_R_7_7 |  |
| ROI-095 | Amyg | Amyg_L_2_1 | mAmyg, medial amygdala |
| ROI-096 | Amyg | Amyg_R_2_1 |  |
| ROI-097 | Amyg | Amyg_L_2_2 | lAmyg, lateral amygdala |
| ROI-098 | Amyg | Amyg_R_2_2 |  |
| ROI-099 | Hipp | Hipp_L_2_1 | rHipp, rostral hippocampus |
| ROI-100 | Hipp | Hipp_R_2_1 |  |
| ROI-101 | Hipp | Hipp_L_2_2 | cHipp, caudal hippocampus |
| ROI-102 | Hipp | Hipp_R_2_2 |  |
| ROI-103 | CAU | BG_L_6_1 | vCa, ventral caudate |
| ROI-104 | CAU | BG_R_6_1 |  |
| ROI-105 | CAU | BG_L_6_5 | dCa, dorsal caudate |
| ROI-106 | CAU | BG_R_6_5 |  |
| ROI-107 | Tha | Tha_L_8_1 | mPFtha, medial pre-frontal thalamus |
| ROI-108 | Tha | Tha_R_8_1 |  |
| ROI-109 | Tha | Tha_L_8_2 | mPMtha, pre-motor thalamus |
| ROI-110 | Tha | Tha_R_8_2 |  |
| ROI-111 | Tha | Tha_L_8_3 | Stha, sensory thalamus |
| ROI-112 | Tha | Tha_R_8_3 |  |
| ROI-113 | Tha | Tha_L_8_4 | rTtha, rostral temporal thalamus |
| ROI-114 | Tha | Tha_R_8_4 |  |
| ROI-115 | Tha | Tha_L_8_5 | PPtha, posterior parietal thalamus |
| ROI-116 | Tha | Tha_R_8_5 |  |
| ROI-117 | Tha | Tha_L_8_6 | Otha, occipital thalamus |
| ROI-118 | Tha | Tha_R_8_6 |  |
| ROI-119 | Tha | Tha_L_8_7 | cTtha, caudal temporal thalamus |
| ROI-120 | Tha | Tha_R_8_7 |  |
| ROI-121 | Tha | Tha_L_8_8 | lPFtha, lateral pre-frontal thalamus |
| ROI-122 | Tha | Tha_R_8_8 |  |

ROI, regions of interest; MPFC, medial prefrontal cortex; DLPFC, dorsolateral prefrontal cortex; SFG, superior frontal gyrus; MFG, middle frontal gyrus; OrG, orbital gyrus; STG, superior temporal gyrus; MTG, middle temporal gyrus; ITG, inferior temporal gyrus; FuG, fusiform gyrus; PhG, parahippocampal gyrus; pSTS, posterior superior temporal sulcus; INS, insular gyrus; CG, cingulate gyrus; Amyg, amygdala; Hipp, hippocampus; BG, basal ganglia; CAU, caudate, Tha, Thalamus.
